# Novel Weigh-in-Motion Pavement Sensor Based on Self-Sensing Nanocomposites for Vehicle Load Identification: Development, Performance Testing, and Validation

**DOI:** 10.3390/s23104758

**Published:** 2023-05-15

**Authors:** Ming Liang, Yunfeng Zhang, Yuepeng Jiao, Jianjiang Wang, Linping Su, Zhanyong Yao

**Affiliations:** School of Qilu Transportation, Shandong University, Jinan 250002, China; ming.liang@sdu.edu.cn (M.L.);

**Keywords:** weigh-in-motion, pavement monitoring, piezoresistive sensor, self-sensing nanocomposites

## Abstract

The development of the transportation industry has led to an increasing number of overloaded vehicles, which reduces the service life of asphalt pavements. Currently, the traditional vehicle weighing method not only involves heavy equipment but also has a low weighing efficiency. To deal with the defects in the existing vehicle weighing system, this paper developed a road-embedded piezoresistive sensor based on self-sensing nanocomposites. The sensor developed in this paper adopts an integrated casting and encapsulation technology, in which an epoxy resin/MWCNT nanocomposite is used for the functional phase, and an epoxy resin/anhydride curing system is used for the high-temperature resistant encapsulation phase. The compressive stress-resistance response characteristics of the sensor were investigated by calibration experiments with an indoor universal testing machine. In addition, the sensors were embedded in the compacted asphalt concrete to validate the applicability to the harsh environment and back-calculate the dynamic vehicle loads on the rutting slab. The results show that the response relationship between the sensor resistance signal and the load is in accordance with the GaussAmp formula. The developed sensor not only survives effectively in asphalt concrete but also enables dynamic weighing of the vehicle loads. Consequently, this study provides a new pathway to develop high-performance weigh-in-motion pavement sensors.

## 1. Introduction

The transportation industry is the backbone of the country’s economic development, among which road transportation is considered to be the lifeline of the economic development of a region and country [1,2]. Additionally, with the continuous development of the road industry, the phenomenon of overloading heavy vehicles has increased [3,4,5]. This has led to a significant reduction in the service life of roads, increased the workload and cost of highway management and maintenance, and also indirectly increased the risk of traffic accidents [6,7,8,9,10]. In this background, road axle load weighing has become a very important part of road infrastructure development [11,12].

At the present stage, the acquisition of highway axle load information is mainly realized by the weighing table and curved plate weighing system at the toll station [13]. However, this parking weighing method not only reduces the efficiency of highway network traffic but also aggravates the problem of congestion at highway toll stations [14]. Therefore, how to accurately obtain the dynamic axle load information of vehicles on highways will be an imminent problem in the construction of smart highway systems. For this reason, some researchers in recent years have carried out optimal design and research on embedded sensing elements based on new sensing materials, such as fiber optic gratings, piezoelectric ceramics, and memory alloys [15,16,17].

Embedded road monitoring and detection devices are sensing devices that are embedded inside the pavement structure in order to obtain data such as pavement structure information and traffic information [18,19]. They can be divided into stress/strain sensors [20], pressure gauges [21,22,23], magnetic induction sensors [24], load cells [25], and vibration sensors [26,27] according to the monitoring object. D Cebon [28] developed a theory for the design of a multi-cell dynamic weighing system with the objective of minimizing errors caused by dynamic axle loads of heavy vehicles traveling at high speeds and validated the system with a wheel load measurement pad with a total length of 38 m. Finally, it was concluded that the error in the dynamic wheel load of the vehicle volume was less than 4% RMS. Alavi S et al. [29] tested Weigh-in-Motion (WIM) sensors via equivalent uniaxial loading on asphalt concrete (AC) and Portland cement concrete (PCC) pavements, and the results showed that the performance of WIM sensors depends on the ability of the data acquisition system to accurately process the raw sensor output. Song G et al. [30] proposed a piezoelectric ceramic smart aggregate that can be used to monitor the health of civil structures, as well as early concrete strength monitoring, impact detection, and structural health monitoring. The monitoring signals were processed by wavelet analysis, and this smart aggregate can be applied for comprehensive monitoring of concrete structures from the initial stage to the whole life cycle. Hou et al. [31] developed an asphalt pavement vehicle axle load monitoring sensor using smart sensing aggregates and demonstrated its linear sensitivity to applied loads, but this sensor had low viability in harsh pavement monitoring environments and was difficult to apply to practical pavement axle load monitoring. Wang and Zhao H [32,33] analyzed the interference signals in the weighing signal of a dynamic weighing system with a quartz crystal load cell as the weighing unit and concluded that there were a large number of interference signals in its weighing signal, including high-frequency noise and low-frequency noise, and used the wavelet transform algorithm to pre-process the weighing data by filtering, and then further processed by the algorithm, and the results showed that the wavelet transform algorithm made the load cell achieve a good weighing effect. Yang H [34,35] designed a stacked array Piezoelectric Energy Harvester (PEH) with a protection package that could improve the performance and lifetime of PEH. A demonstration project was also conducted to test the field performance. It was found that the obtained piezoelectric energy could successfully illuminate LED signs under real vehicle loads. The electrical response of the stacked piezoelectric units was also tested under different temperatures and loads, and it was found that the ambient temperature had a significant effect on the piezoelectric power generation, and the electrical power generated by the piezoelectric units increased with the increase in load. Xiong H et al. [36] developed a Piezoelectric Weigh-in-motion (P-WIM) based on piezoelectric material, which consisted of several piezoelectric material discs capable of generating characteristic voltage output from passing vehicles, and by analyzing the voltage generated by the P-WIM, the axle load of the vehicle could be determined. Zhao [37] proposed a multi-cell dynamic weighing system based on an array of force sensors combined with vibration sensors based on piezoelectric ceramics to achieve dynamic weighing of pavement axle loads, but other related studies have shown that piezoelectric ceramics are less resistant to high temperatures and may show phenomena such as data instability in summer [38]. The working principle of the fiber optic load sensing system is the change in reflected light intensity within the fiber when the vehicle passes through the fiber optic sensor, the compressive stress value is calculated based on the magnitude of the intensity change, and then the measured dynamic compressive stress is used to predict the true axle weight of the vehicle. However, the monitoring accuracy of this type of sensor and piezoelectric sensor is susceptible to the harsh service environment inside the pavement [39,40].

Within the harsh monitoring environment of asphalt pavements, the author’s team conducted a series of related studies in the development of new sensors for pavements [41,42,43]. Smart-material-based tensile strain sensing elements have been developed, and the high accuracy and viability of the sensing elements have been verified. Moreover, the study of the cracking and deformation of materials at the nanoscale helps to uncover the microscopic deformation and working mechanism of nanomaterial sensors and improve the performance of sensors [44]. Based on the above research background and the current status of research [45,46], the authors found that the types of road vehicle axle load sensors available at this stage are diverse and the materials used vary, but almost all suffer from unstable service performance or even failure in harsh environments. The pre-embedded technology and high-temperature rut test are used to simulate the harsh environment; this paper intends to design and prepare an embedded piezoresistive sensor based on smart materials to solve the current problems of low viability and poor stability of asphalt pavement vehicle axle load monitoring elements and to investigate the stress-resistance response characteristics of smart materials under compressive stress, which provides a new idea for weigh-in-motion of asphalt pavement vehicles.

## 2. Development of Piezoresistive Sensors Based on Self-Sensing Nanocomposites

### 2.1. Preparation of Self-Sensing Nanocomposites

The smart materials chosen in this paper are an epoxy resin/multi-walled carbon nanotube (MWCNT) composite. The multi-walled carbon nanotubes were provided by Nanjing Jicang Nanotechnology Co., Ltd. (Nanjing, Jiangsu Province, China). and were prepared by high-temperature cracking of acetylene catalyzed by a nickel-based catalyst. The multi-walled carbon nanotubes have a purity of 95 wt%, an average diameter of 10–20 nm, a length of 30–100 μm, a specific surface area greater than 165 m^2^/g, and excellent electrical conductivity of more than 1250 s/cm. The epoxy resin is bisphenol A epoxy resin with an epoxy value of 0.48–0.54 eq/100 g and viscosity of 12,000 mPa·s, supplied by Nantong Star Synthetic Materials Co., Ltd. (Nantong, Jiangsu Province, China). The epoxy resin curing agent is an amine curing agent, and dimethylformamide (DMF) is selected as the dispersant.

For the formed smart materials to form an effective conductive network, the multi-walled carbon nanotubes should be uniformly dispersed in the epoxy polymer matrix, and the aforementioned studies have shown that the degree of dispersion plays a key role in the stress–resistance response [41,42,43]. Therefore, in order to avoid the phenomenon of non-uniform dispersion due to large van der Waals forces caused by the large specific surface area and small nanoparticle size, in this study, multi-walled carbon nanotubes were first ground in an agate mortar for 30 min and then mixed thoroughly with the dispersant DMF with mechanical stirring for 20 min, then sonicated for 1 h with an ultrasonic disperser (UH450 Oulior). After that, epoxy resin was added to the mixed DMF/carbon nanotube suspension, and sonication was continued for 1 h. Solvent evaporation was carried out at 80 °C in a vacuum-drying oven for 1 h. Finally, the curing agent was added and stirred for 10 min to produce the composite smart material.

### 2.2. Molding and Encapsulation Structure Design

**Figure 1:**
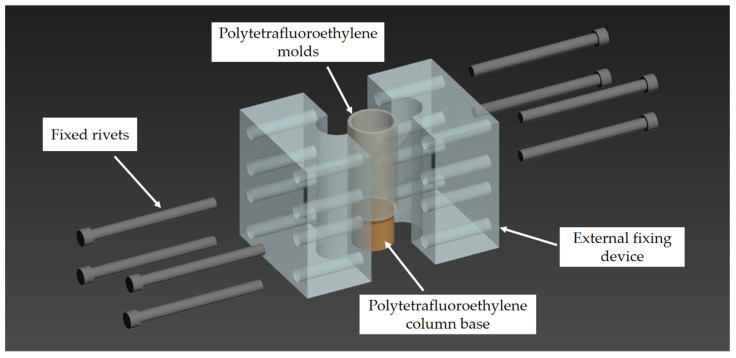
Schematic diagram of integration mold for sensing material.

Epoxy resin, the polymer matrix of the composite material in this sensor, has a very high bonding strength, which makes it difficult to de-mold if a metal mold is used. However, epoxy resin does not have such a high bond strength for Teflon-type materials, so it is easy to de-mold. In addition, epoxy-resin-based smart materials are viscous-fluid before curing, and smart devices with certain structural characteristics can be prepared by integrated casting in the mold. After indoor tests to fully verify the molding effect and ease of de-molding of the smart material, the preferred smart material molding inner mold was determined to be a Teflon tube (14 mm high, 20 mm diameter) with the bottom end sealed by a Teflon cylindrical plug. The conductors are inserted inside the mold through the top and bottom through-holes and buried inside the material with smart material curing. In addition, in order to prevent the Teflon tube from shifting after the material is poured and while waiting for the epoxy resin to cure, resulting in changes in sensor size and form, the Teflon tube is supported on the outside by two semicircular arc fixing brackets and is fixed in the middle using rivets. The overall design of the mold is shown in Figure 1. The smart materials and the mold are placed in the oven after the casting is completed, and the sample is de-molded after curing at 80 °C for 2 h to produce the smart materials’ inner core of the axle load sensor. By enlarging the inner diameter of the mold and following the same casting method, an epoxy resin/anhydride system was poured around the smart materials’ inner core as a polymer encapsulation structure, thus significantly improving the high-temperature resistance and reducing the humidity sensitivity of the sensor.

**Figure 2:**
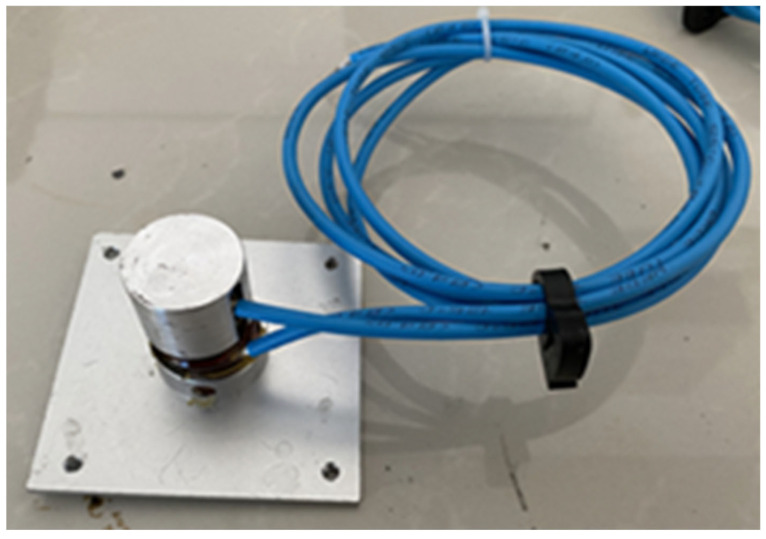
Piezoresistive sensor based on composite smart materials.

To ensure the viability of the cylindrical smart sensor, a protective cap for the metal encapsulation of the sensor is designed. The cap is bonded to the sensor through the top surface only, and the inner diameter is larger than the outer diameter of the sensor. This structure can effectively protect the piezoresistive sensor, does not limit the lateral deformation of the sensor, and will not affect the monitoring accuracy. At the same time, considering the influence of the lower support of the axle load sensor on the monitoring stability, a square enlarged base with a side length of 7 cm is designed. The sensor with the added protective cap cover and base is shown in Figure 2.

The epoxy/MWCNT smart material sensor works by predicting the actual vehicle weight by reading the change in resistance of the sensor. When the sensor receives a vehicle load, the MWCNT inside the sensor rearranges to form a new electrical path, and the internal resistance of the sensor changes. Data acquisition and accuracy are achieved by connecting the sensor wiring to an external data collector channel and inputting the calibration coefficients for each sensor into the corresponding channel. The durability and viability of the sensors can be ensured by wired data transmission.

## 3. Dynamic Response Test for Compressive Stresses

In order to investigate the stress–electrical response law of piezoresistive sensors of composite smart materials, the stress–electrical response of the sensor is calibrated by using a universal testing machine. The peak load is set to 15 kN, the preload force is set to 10 N when loading, and the loading starts from 0 kN according to a certain loading rate, which can cover the vehicle overload of 400% according to the standard load of 0.7 MPa to verify the change law of sensor resistance response under different loading forces. Additionally, changing the magnitude of the loading rate can be used to explore the effect of different loading rates on the stress–electrical response law of the sensor (the loading rates are 20 N/s, 80 N/s, 140 N/s, 200 N/s, 260 N/s)

**Figure 3:**
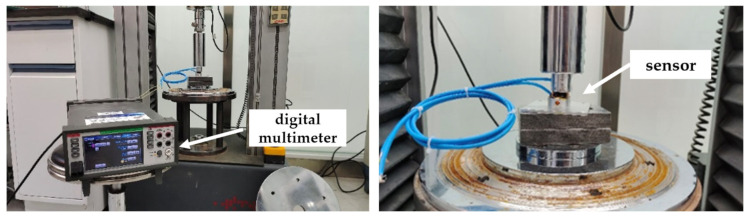
Calibration test via universal testing machine.

**Figure 4:**
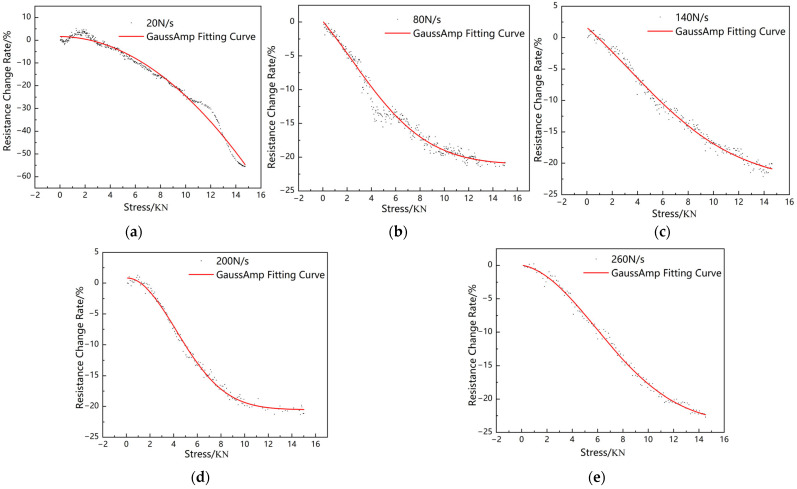
Fitting diagram of compressive stress–resistance response at different loading rates. (**a**) 20 N/s, (**b**) 80 N/s, (**c**) 140 N/s, (**d**) 200 N/s, and (**e**) 260 N/s.

As shown in Figure 3, the sensor resistance signal is recorded throughout the loading process by KEITHLEY DAQ6510 digital multimeter. According to the loading of the MTS tester, when the loading force reaches 100 N, the sensor starts to show resistance changes; when the loading force reaches 15 kN, the sensor resistance changes gradually tend to slow down, indicating that the upper and lower limits of the load monitored by the sensor are 15 kN and 100 N, respectively. Figure 4 shows the stress–resistance response at different loading rates. It can be seen in the figure that the data of the axle load sensor based on the composite smart material have a large dispersion and exhibit randomness at lower loading rates. However, as the loading rate increases, the dispersion of the data gradually decreases and tends to a stable negative correlation. When the loading rate is small, the data are more dispersed, and as the loading rate increases, the dispersion of the data decreases. Smaller loading rates take more time to load to a given load than larger loading rates, and there is a certain creep effect on the material inside the sensor that affects the subsequent loading and, therefore, a greater dispersion of data. As the loading rate increases, the creeping effect of the above materials mentioned decreases, and the dispersion of the data decreases. When the loading rate is greater than 140 N/s, the data fit is greater. Obviously, the loading rate of the vehicle axle load acting on the road is greater than this rate. Therefore, the sensor can maintain stable sensing characteristics when used to monitor the road axle load. By performing a nonlinear fit to the images for loading rates of 140 N/s and above, the results show that the fitted response curve conforms to the GaussAmp formula [47,48,49], and the fitted curve expression is

(1) $$y=y_{0}+Ae^{-\frac{{\left(x-x_{c}\right)}^{2}}{2w^{2}}}$$

The average value of R^2^ for the three fitting results is 0.99331, indicating that the stress–resistance response relationship of the developed axle load sensor conforms to the GaussAmp formula. It can be shown that the sensor developed in this paper can reflect the sensing of stress by its resistance change. Thus, in practical applications, the axle load of a vehicle on the road can be monitored indirectly by monitoring the resistance of the piezoresistive sensor according to the GaussAmp formula.

## 4. Validation of the Developed Sensor for Vehicle Load Identification

### 4.1. Embedment and Dynamic Weighing in Asphalt Concrete Slab

**Figure 5:**
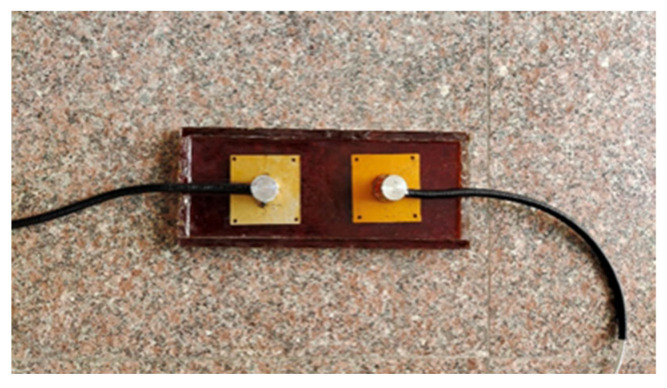
Epoxy base for positioning.

In order to simulate the working environment of the axle load sensor inside the asphalt concrete, the sensor is embedded in the rutting slab prepared from AC-13 mix for a compressive stress sensing test. Since the axle load sensor is a columnar structure with a small diameter, it is difficult to ensure that it is stable and does not shift if it is directly buried in the rutting slab, so a base will be designed to assist the axle load sensor in fixing its position. The difference between the modulus of elasticity of the base and the modulus of elasticity of the asphalt mixture may lead to stress concentration in the asphalt mixture. Therefore, instead of using a metal base similar to the piezoelectric quartz sensor, an epoxy resin base with a similar modulus of elasticity to that of the asphalt mixture is used, as shown in Figure 5.

**Figure 6:**
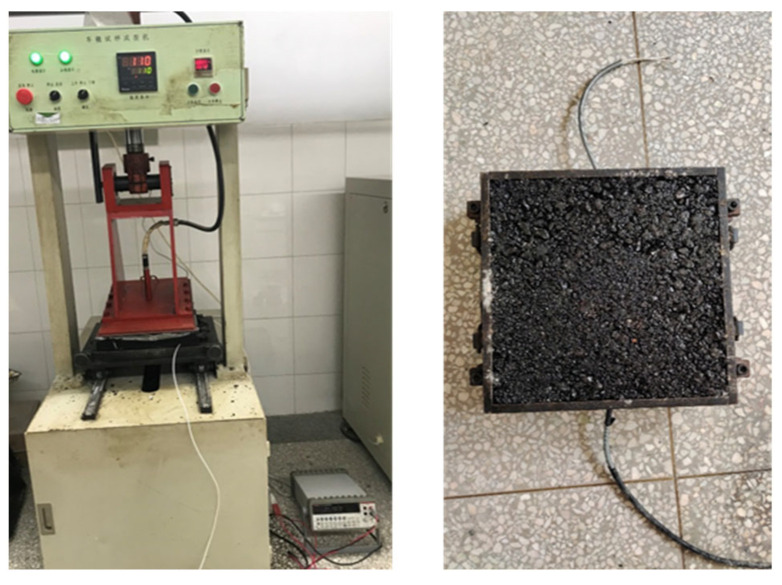
The embedment of piezoresistive sensors and formation of rutting slab.

AC-13 mixture mixes are shown in Table 1:

The burial depth of the axle load sensor in asphalt concrete is a key factor affecting the monitoring accuracy. Studies have shown that axle load sensors embedded in the surface layer of the pavement have the best monitoring effect [34,35], but burial in the surface layer of the pavement with a serious disease is not conducive to the long-term service of the sensors [50,51,52,53]. Therefore, in this paper, the sensor base is embedded at the bottom of the rutting slab, and the top of the sensor is 2 cm from the surface layer of asphalt concrete after burial. The embedded sensor is shown in Figure 6 after the asphalt concrete is compacted and shaped. The embedded sensors have a corresponding piezoelectric response after being subjected to a high-temperature environment and heavy wheel compaction. It is verified that the developed sensor can withstand the harsh environment during asphalt pavement construction. In addition, compared to the excavation-embedded axle load sensors, the direct burial of the smart sensor during construction reduces the perturbation of the sensor to the dynamic response of the pavement. It also reduces the damage to the pavement structure and offers the possibility of significantly simplifying the axle load sensor burial process.

**Figure 7:**
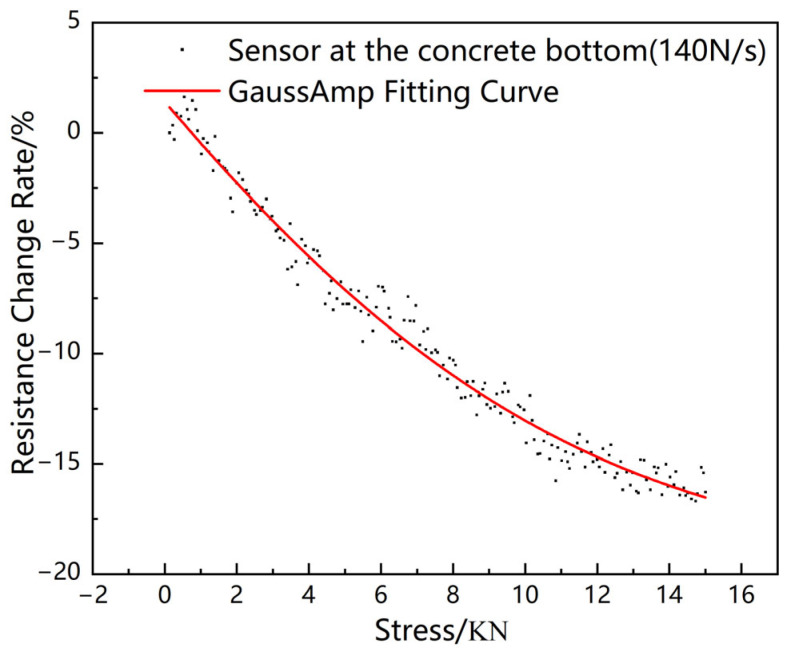
Compressive stress response of sensors in asphalt concrete.

According to the previously described sensor response characteristics, the asphalt concrete compressive stress sensing test was loaded at a loading rate of 140 N/s. The experimental results are shown in Figure 7. The response characteristics of the sensor buried in the asphalt concrete still conform to the GuessAmp fitting equation described previously. The calibration fitting results before and after the sensor was buried in the rutting slab are as follows.

Sensor response fitting curve before burial:

(2) $$y=-0.23+0.29e^{-\frac{{\left(x+5.24\right)}^{2}}{2\times 8.49^{2}}},$$

Sensor response fitting curve after embedding:

(3) $$y=-0.19+0.22e^{-\frac{{\left(x+5.26\right)}^{2}}{2\times 8.5^{2}}},$$

Comparing the sensor response fitting curves before and after burial, it can be seen that the response data of the axle load sensor after burial in the rutting slab are about 0.77 times that before burial according to the burial depth and loading rate selected in the test. Therefore, for practical engineering applications of axle load sensors, the results of the indoor calibration of the sensor can be multiplied by the corresponding correction factor to back-calculate the actual axle load of the vehicle

### 4.2. Applications for Weigh-in-Motion

**Figure 8:**
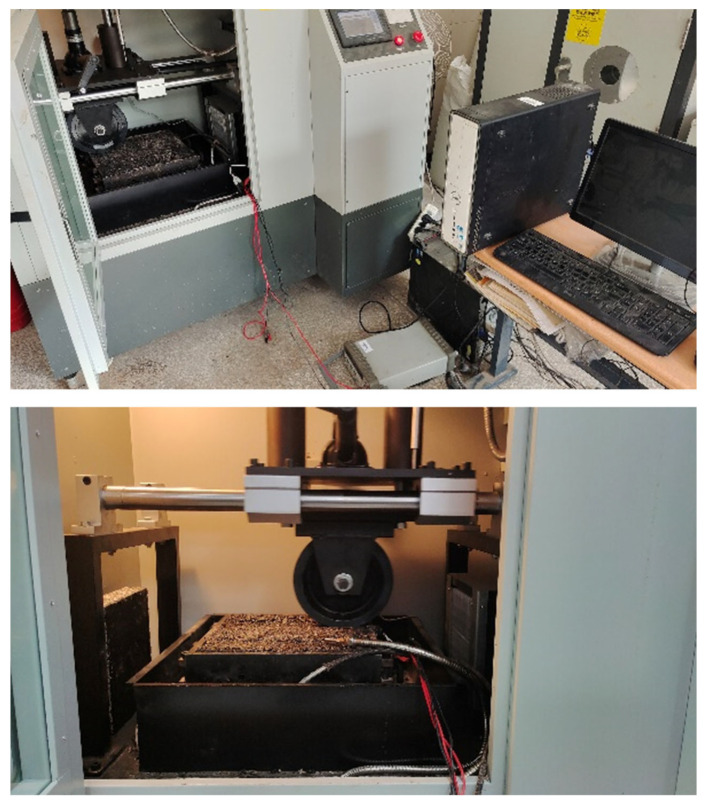
Rutting experiment for axle load sensor.

The rutting apparatus was used to simulate the traveling load and to investigate the load-electric response law of the developed sensor under the traveling wheel load. The test parameters were set according to the relevant specifications for asphalt mixture rutting tests in China (JTG E20-2011-T 0719-2011). As shown in Figure 8, the temperature of the asphalt concrete specimen with the embedded sensor was stabilized at 60 ± 0.5 °C during the test, and the test wheel travel track was located directly above the sensor in the rutting slab. Three parallel experiments were conducted, and the average of the three groups was taken for performance analysis.

**Figure 9:**
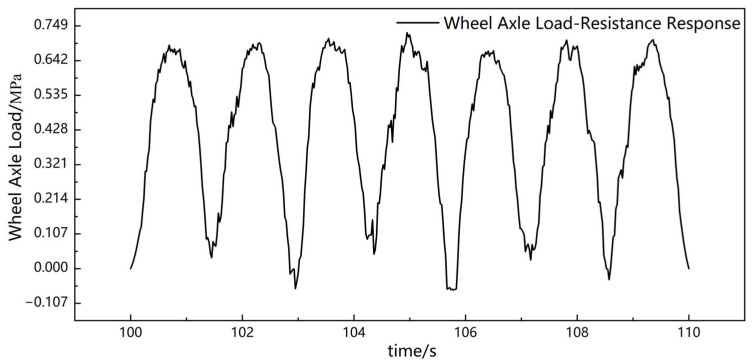
Compressive stress–resistance response during rut experiment.

The sensor has a significant resistance drop when subjected to wheel load, and the drop amplitude is stable at the same level. In this paper, the experimental results between 100 and 110 s during the experiment were selected, and the axle load conversion was carried out according to the response fitting curve (3) of the sensor after embedding, as shown in Figure 9.

According to the indoor calibration results, it can be back-calculated that the wheel load acting on the top of the rutting slab is 0.68–0.74 MPa, which is near the actual load (0.75 MPa) action level. It can be shown that the composite smart material piezoresistive sensor developed in this paper can monitor the vehicle load in real-time within the pavement, and the monitoring of the axle load magnitude is also accurate. At the same time, the time course curve of the axle load can also be used to obtain the dynamic vehicle speed information by analyzing the relative positions of the peaks/valleys. It provides a possibility to develop high-performance weigh-in-motion pavement sensors for accurate, long-term, and stable acquisition of traffic flow information.

## 5. Conclusions and Discussion

In this paper, a new embedded piezoresistive sensor based on self-sensing materials is developed to address the problems of existing axle load weighing. It is calibrated by indoor experiments, and the implementation of this new sensor for dynamic load monitoring is investigated with the following conclusions.

(1) The resistance of the novel piezoresistive sensors is negatively correlated with the magnitude of the external load applied to it, and the relationship satisfies the GaussAmp formula. Moreover, the mean value of the goodness of fit of the GaussAmp formula at the pavement vehicle load loading frequencies (140 N/s, 200 N/s, 260 N/s) reaches 0.99331, indicating that there is a good correlation between the pressure and the rate of change of the resistance at these frequencies, and the pressure applied can be back-calculated from the rate of change of the resistance of the sensor.
(2) Comparing the sensor response fitting curves, it can be seen that the response data of the axle load sensor after burial in the rutting slab are about 0.77 times that before burial according to the burial depth and loading rate selected in the test (the top of the sensor is 2 cm from the surface layer of asphalt concrete after burial, and the loading rate is 140 N/s).
(3) The rutting experiment verifies that the sensor has a high sensitivity to the dynamic load, and the vehicle load and speed information can be obtained by analyzing the peak/trough magnitude and frequency of the output electrical signal.

Finally, the developed novel piezoresistive sensor based on nanocomposites is competent in vehicle weight and speed detection. It opens up a new territory to develop high-performance weigh-in-motion pavement sensors for long-term, accurate, and low-cost monitoring.

## Figures and Tables

**Table 1:** AC-13 mix ratio design.

Raw Materials	Specification	Matching Ratio (%)	Quality (g)
Asphalt	Qilu AH-70	4.25	559
Aggregates	13–16 mm	6.00	746.4
	10–13 mm	14.00	1741.8
	5–10 mm	34.00	4230
	3–5 mm	17.00	2115
	0–3 mm	26.00	3234.6
Mineral powder	0.075 mm	3.00	373.2

## Data Availability

All data are available in the manuscript.
